# Experimental Models to Study the Role of Microbes in Host-Parasite Interactions

**DOI:** 10.3389/fmicb.2016.01300

**Published:** 2016-08-23

**Authors:** Megan A. Hahn, Nolwenn M. Dheilly

**Affiliations:** School of Marine and Atmospheric Sciences, Stony Brook UniversityStony Brook, NY, USA

**Keywords:** holobiont, symbioses, microbiome, microbe, mutualism, experimental models

## Abstract

Until recently, parasitic infections have been primarily studied as interactions between the parasite and the host, leaving out crucial players: microbes. The recent realization that microbes play key roles in the biology of all living organisms is not only challenging our understanding of host-parasite evolution, but it also provides new clues to develop new therapies and remediation strategies. In this paper we provide a review of promising and advanced experimental organismal systems to examine the dynamic of host-parasite-microbe interactions. We address the benefits of developing new experimental models appropriate to this new research area and identify systems that offer the best promises considering the nature of the interactions among hosts, parasites, and microbes. Based on these systems, we identify key criteria for selecting experimental models to elucidate the fundamental principles of these complex webs of interactions. It appears that no model is ideal and that complementary studies should be performed on different systems in order to understand the driving roles of microbes in host and parasite evolution.

## Introduction

**FIGURE 1:**
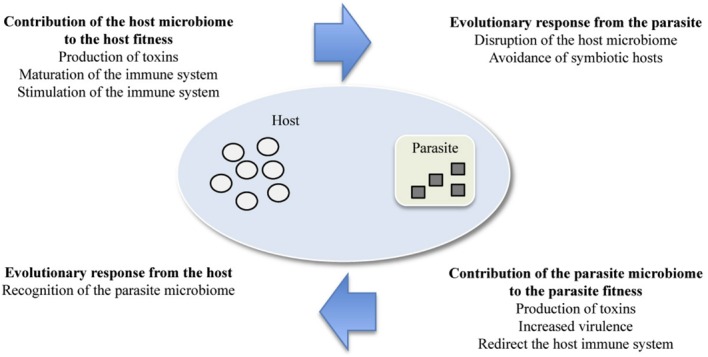
**Contribution of the host and parasite microbiome to the host and parasite fitness and evolutionary responses expected from the parasite and host, respectively.** The smaller symbols represent the microbes; the shape and color indicate whether it is associated with the host (white circle) or parasite (gray square).

The term ‘symbiosis’ (from Greek “living together”) was first coined by Albert Bernhardt Frank in 1877, when he described the relationship between fungi and algae in lichens, and was then used to define “the living together of unlike organisms” by de Bary (1879) and Theis et al. (2016). The modern definition of symbiosis encompasses all beneficial, neutral, and harmful long-term and intimate relationships, which correspond to a continuum of mutualistic, communal, and parasitic symbiosis (Bronstein, 2009; Cheng, 2012). Symbionts can contribute to phenotypic variations and thus to evolutionary innovation in their hosts (Margulis, 1991). Recent developments in molecular techniques have propelled a vast expansion of our knowledge regarding the presence, diversity, and abundance of non-cultivable microbes. It has revealed that most often, organisms are not associated with one single symbiont, but with a myriad of microbial symbionts that can independently or synergistically participate in their physiology and evolution (Rosenberg and Zilber-Rosenberg, 2011). The recognition of this inherent complexity of organisms led to the concept of the holobiont that integrates the host organism and all its associated microbes (Margulis, 1991). Two divergent opinions have recently risen. Some argue that the term holobiont and the concepts associated to it can lead to misunderstanding of evolutionary concepts in host-associated microbes (Moran and Sloan, 2015; Douglas and Werren, 2016). Others maintain that this new concept is necessary to embrace the pluralistic attributes of any plants and animals, whose phenotype result from multilevel selection on the host genome, the microbe genomes, and the hologenome (Bordenstein and Theis, 2015; Theis et al., 2016). This complexity and its implications has renewed interest in symbionts and is revolutionizing many fields of biology including parasitology and immunology. Of interest in this review, host-parasite interactions are now being re-considered in light of the microbiome (Dheilly, 2014). This has led to a better understanding of the pluralistic role of microbes in host defense and to new theories of parasite virulence strategies driven by their interaction with microbes (Dheilly et al., 2015b). Host and parasite microbiomes contribute to host and parasite fitness and impose new selection pressures on parasites and hosts, respectively (**Figure 1**). Alteration of any partner may cause a chain of responses in those remaining and change the outcome of infection.

## Host Defensive Symbionts

Scientists have long recognized and accepted that mixed infection is the rule in natural environments and that microbes in the same host can directly and indirectly interfere with each other. Concomitant immunity was shown in Schistosomes (blood flukes) as early as 1969 and facilitation in 1971 (Kloetzel et al., 1971, 1977). We now know that resistance, competition, and facilitation can result from direct and indirect interactions among protozoa, helminthes, viruses, and bacteria (Cox, 2001). Thus, the net outcome of the host-parasite interaction can vary greatly in presence of other microbes, from harmful to neutral and beneficial. In particular, the concept of defensive mutualism led to the discovery of microbes acting in defense to parasites in many taxa, including fungi, mollusks, insects, plants, and vertebrates (Clay, 1988; White and Torres, 2009). Microbial symbionts are necessary for maturation of the immune system during early-life (Gollwitzer and Marsland, 2015) and later protect the host against pathogens via immune activation (Hall et al., 2008; Benson et al., 2009), production of antimicrobial factors, competition for resources or by limiting parasite adhesion to host cells (Cash, 2006; Kamada et al., 2012; Buffie and Pamer, 2013). Most importantly, microbe-mediated protection can evolve rapidly due to their large population size and short generation times, thus participating in host adaptation (King et al., 2016). In fact, symbionts may take over the evolutionary arms race against parasites, and alleviate the selection pressures on the host (Kwiatkowski et al., 2012), as demonstrated by the reduction in *Drosophila melanogaster* resistance to *Drosophila* C virus when associated with the defensive symbiont *Wolbachia* (Martinez et al., 2016a). The inter-regulation between microbiome and immune system has led to redefinition of this new unit that integrates host and microbe-encoded defenses into a single “holo-immunome” (Dheilly, 2014) or “microimmunosome” (Dietert, 2016).

A number of organismal systems were developed to study the role of defensive symbionts in the ecology and evolution of hosts and parasites. Many insects harbor microbial mutualists and studies of their role in defense to parasites have long been successful. Aphids have provided the most remarkable example of insect-associated defensive symbionts (see Oliver et al., 2014 for detailed review). In *Acyrthosiphon pisum*, the facultative symbionts *Hamiltonella* and *Serratia* provide defense against the parasitic wasp *Aphidius ervi* (Oliver et al., 2003, 2005) whereas *Regiella* provides resistance to the fungus *Pandora neoaphidis* (Scarborough et al., 2005). Because aphids have a parthenogenetic reproduction, clonal lines of aphids can be maintained indefinitely and the microbiome is manipulated by a combination of antibiotic treatments and micro-injection of symbionts. Aphids are thus ideal experimental models to understand the impact of defensive symbionts on the evolution of their hosts and parasites. For instance, it is employed to study the cost of defensive symbionts (Vorburger et al., 2013; Polin et al., 2014; Cayetano et al., 2015), their population dynamics (Oliver et al., 2008), their role in parasitoid community composition (Rothacher et al., 2016), the specificity of symbiont-associated defense (Martinez et al., 2016b), and to demonstrate that host symbiont’s role in defense imposes new selection pressure on parasites (Oliver et al., 2012). Another biological system worth mentioning is the role of Wolbachia against viruses. *Wolbachia* is a reproductive parasite of the fruit fly *Drosophila melanogaster* (Fry et al., 2004) but it also imparts resistance to *Drosophila* C viruses, Cricket Paralysis Virus, Flock house virus, Nora virus, and West Nile Virus by reducing the viral load (Hedges et al., 2008; Teixeira et al., 2008; Osborne et al., 2009; Glaser and Meola, 2010). Knowledge gained from studying this organismal system has led to the discovery of a *Wolbachia*-induced resistance to many human parasites including Dengue virus, Chikungunya virus, Yellow fever virus, West Nile Virus, and malaria parasites in mosquitoes (Moreira et al., 2009; Bian et al., 2010; Van den Hurk et al., 2012). Clearly, integrating the role of defensive symbionts into our understanding of vector competence has implications for the control of disease transmission. For example, *Wolbachia*-infected mosquitoes have been released in natural environments as a preventative measure to limit the prevalence of Dengue virus (Hoffmann et al., 2011; Iturbe-Ormaetxe et al., 2011). The diversity of insects carrying defensive symbionts provides opportunities to answer many including the extent of host system reliance on defensive symbionts, the role of competition among symbionts, or the potential co-diversification of defensive symbionts with parasites.

In vertebrates, emerging evidence shows that microbes inhabiting many sites in the body have a role in maintaining health even though few studies examine the direct role of defensive symbionts in resistance against parasites. Promising experimental systems include the red-back salamander, *Plethodon cinereus* that hosts cutaneous, antifungal metabolite-producing bacteria, which prevent infection by pathogenic fungi *Batrachochytrium dendrobatidis* (Brucker et al., 2007, 2008). In birds, *Upupa epops* and *Phoeniculus purpureus*, the uropygial glands house symbiotic bacteria that secrete protective antimicrobial substances providing protection to the feathers and eggs (Martín-Platero et al., 2006; Ruiz-Rodriguez et al., 2009; Martín-Vivaldi et al., 2010, 2014; Ruiz-Rodríguez et al., 2013). Further studies of these tractable vertebrate biological systems are necessary to characterize the significance of the increased resistance and test ecological and evolutionary theories that are mostly developed on invertebrate systems.

## Parasite Disruptive Strategies

Many parasites share the same environment as host-associated microbes, including defensive symbionts. Interestingly, parasites from all major phyla, including helminthes, viruses, bacteria, and fungi modify their host microbiome. For example, parasitic helminth infection results in significant modifications of gut microbiome in humans, pigs, cattle, hamsters, goats, and sheep (Nicholls, 1987; Li et al., 2011, 2012, 2016; Wu et al., 2012; Plieskatt et al., 2013; Cantacessi et al., 2014; Lee et al., 2014). But it is unknown if the modifications in the composition of the host microbiome are beneficial for the parasite, a defense mechanism of the host, or by-products of the pathology. It was proposed that parasites can directly modify their microbial environment to make it more favorable, or to exercise immune-modulatory activity and reduce the host resistance to infection, a process called the disruptive strategy (Dheilly et al., 2015b). In helminths, evidence suggests that alteration of the host microbiome indeed participates in parasite strategies to modulate the host immune system (Plieskatt et al., 2013). This theory is also supported by the observation that hatching by the nematode *Trichuris muris* is dependent on an abundant microflora (Hayes et al., 2010; Kane et al., 2011; Kuss et al., 2011) but the beneficial role of parasite-associated disruption of the host microbiome remains to be experimentally tested in a tractable system. Trophically transmitted parasites such as cestodes, are of interest because they have the potential to interact with microbes associated with both definitive and intermediate hosts. *Triaenophorus nodulosus* and *Eubortium rugosum* have been found to physically interact with many gut bacteria of their intermediate hosts, *Esox Lucius* (pike) and *Lota lota* (burbot), respectively, (Izvekova and Lapteva, 2004). In the rat, *Hymenolepis diminuta* infection results in a significant modification of the gut microbiome composition and a stronger stability in response to a mild inflammatory challenge (McKenney et al., 2015). The cestode parasite, *Schistocephalus solidus*, appears as a model of choice to investigate cestode parasite interactions with host gut microbes because all parasite stages can be cultured *in vitro* by mimicking either the avian intestinal environment, or the copepod or the stickleback fish body cavity (Smyth, 1990; Jakobsen et al., 2012), which allows rigorous functional experiments. Of interest, the highly specific second intermediate host of *S. solidus*, the threespine stickleback has a broad geographical variation (Hagen and Gilbertson, 1972) and has become a model species in biology for both ecological and evolutionary studies of the interaction (Barber and Nettleship, 2010).

While most microbiome disruptions by parasites result in subtle changes with unknown consequences for the outcome of infection, some have spectacular consequences. *Vibrio shiloi*, a bacterium infecting the coral host *Oculina patagonica*, produces toxins specifically targeting the coral’s symbiotic zooxanthellae (Kushmaro et al., 1997, 1998, 2001). Following the temperature-dependent attachment to the coral surface, *V. shiloi* infiltrates the tissue and multiplies rapidly (Kushmaro et al., 1998; Toren et al., 1998; Banin et al., 2000). The bacterium then releases photosynthesis-inhibiting toxins and lyses zooxanthellae, eventually leading to coral bleaching and death (Ben-Haim et al., 1999). In another key example, the microsporidian parasite *Paranosema locustae* relies on gut acidification of its locust host (*Locusta migratoria manilensis*) and increased production of reactive oxygen species to modify the gut microbial community and insure its own development (Shi et al., 2014). Because the locust microbiome is involved in producing pheromones responsible for the locust swarming behavior, infection by *P. locustae* prevents swarming (Dillon et al., 2000; Shi et al., 2014). The adaptive value and specificity of *V. shiloi* and *P. locustae* for their host-associated microbes remain to be demonstrated but the obvious phenotypic changes associated with the microbiome disruption increases the value of these organismal systems. In addition, they provide the opportunity to study evolutionary trade-offs of parasite induced disruption of the host microbiome.

## Parasite Biological Weapon Strategies

The presence of microbes associated with hosts has been demonstrated in many systems, but the presence of microbes associated with parasites has been tested less frequently. For example, *Wolbachia* is an obligate bacterial symbiont of parasitic filarial nematodes (Kozek, 1977; Hoerauf et al., 1999). *Wolbachia* incite inflammatory reactions in the human host and it has been implicated in many debilitating diseases (Taylor and Hoerauf, 1999). For instance, *Wolbachia*-induced inflammation is responsible for the loss of vision and blindness following *O. volvulus* infection (Francis et al., 1985; Hall and Pearlman, 1999; Saint André et al., 2002) and for the inflammation of lymphatic vessels following *W. bancrofti* infection (Dreyer et al., 2000; Hoerauf et al., 2003). Similarly, the trematode *Opisthorchis viverrini*, also known as liver fluke, often leads to cholangiocarcinoma (Braconi and Patel, 2010). Recent studies demonstrated that *O. viverrini* is associated with a complex microbiome (Plieskatt et al., 2013) and that some of these bacteria are transmitted to the host and present in abundance in tumors, calling for more basic experiments to determine their contribution in cancer development (Chng et al., 2016; Osborne and WegenerParfrey, 2016). Other parasites are associated with viruses, such as *Trichomonas vaginalis*, a protozoan parasite that infects the human vagina (Goodman et al., 2011). Approximately half of all clinical samples carry a dsRNA virus called *Trichomonavirus* that is recognized by the human immune system and causes an inflammatory response (Fichorova et al., 2012) responsible for increased susceptibility to sexually transmitted diseases (Van Der Pol et al., 2008), infertility (Grys, 1972; Grodstein et al., 1993), miscarriage (Minkoff et al., 1984), pre-term delivery (Hardy et al., 1984; Minkoff et al., 1984), low birth weight (Hardy et al., 1984), and cervical cancer (Gram et al., 1992; Kharsany et al., 1993). The presence of the *Trichomonavirus* also explains why symptoms can worsen upon successful antibiotic treatment: the dying parasite releases its viral load within the human vagina (Fichorova et al., 2012). Notably, parasites from various phyla are associated with viruses, including trematodes and parasitic protozoans but their impact on the host has been rarely investigated (Ip and Desser, 1984; Wang and Wang, 1991; Ives et al., 2011). Clearly, the significance of parasite-virus and parasite-bacteria associations for treatment of the diseases is evident. But, what is the significance of the phenomenon for the parasites themselves? It has been proposed that parasites can utilize symbionts to increase their virulence, a process called the biological weapon strategy (Dheilly et al., 2015b). Clearly, further experimental studies are needed to determine if symbiotic bacteria or viruses and associated inflammation are beneficial for the parasite.

A promising tractable experimental system involves the parasitoid wasp, *Dinocampus coccinellae* that infects ladybeetles and carries a RNA virus, *D. coccinellae* paralysis virus (DcPV; Dheilly et al., 2015a). In adult wasps, DcPV is stored in the oviduct. The wasp injects its eggs into the body cavity of the ladybeetle and the growing larvae feed off the ladybeetle’s fat body. During larval development, the virus replicates actively and is transmitted to the ladybeetle. DcPV then targets the ladybeetle nervous tissue. When later a single mature larva egresses from the host, DcPV replication in the ladybeetle nervous system results in the induction of antiviral immune responses and damage to the nervous tissue associated with ladybeetle paralysis. The larva stills between the legs of the immobilized ladybeetle and spins a cocoon within which it pupates into an adult wasp. The ladybeetle appears crouched protectively over the cocoon of the wasp and makes erratic movements efficiently deterring predators (Maure et al., 2011). Upon larva pupation, clearance of DcPV and nervous system regeneration results in the ladybeetle recovery of normal behavior (Dheilly et al., 2015a). Therefore, it appears that *D. coccinellae* uses DcPV as a biological weapon to manipulate its ladybeetle host and protect its progeny from predation (Maure et al., 2011; Dheilly et al., 2015a). This organismal system appears ideal to investigate the role of associated viruses in the evolution of parasites because of the short generation time of host and parasite, the broad geographic range of the parasite that infect ladybeetle species on all continents, and because of the possibility to track variations in virus genome in experimental evolution experiments. Most interestingly, ladybeetles are also associated with a diverse array of bacteria including multiple members of the genus *Flavobacteria*, *Rickettsia*, *Spiroplasma*, and *Wolbachia*, that may be involved in ladybeetle defense against the parasitoid (Hurst et al., 1994, 1997; Weinert et al., 2007; Elnagdy et al., 2013). Using techniques developed previously to study the defensive role of aphid symbionts, the microbiome of ladybeetles could be manipulated using a combination of antibiotic treatments and microinjections. Thus, this organismal system provides an opportunity to investigate the possibility for direct interactions between host-associated microbes and parasite-associated microbes within the body cavity of the host.

## Conclusion

Understanding the role of microbes in host and parasite ecology and evolution is important for both basic and applied biology, as so many disease-causing organisms carry their own microbes or interact with host-associated microbes. Empirical studies of host-parasite-microbes interactions at a mechanistic and evolutionary level can help us understand the ecological circumstances where symbiosis benefits host or parasite fitness. The current review of experimental systems enabling studies of host-parasite-microbe interactions has allowed us to identify key criteria to improve upon and further study these complex interactions (**Box 1**, Supplementary Table S1). We would like to emphasize that it is now crucial to investigate the role of community of microbes as well as individual microbial symbionts. Indeed, microbes may independently, synergistically, or antagonistically impact and participate in the physiology and evolution of their hosts Therefore, it is of particular importance to develop models where it is possible to intimately manipulate the host and parasite microbiomes to alter their composition. We expect that future research project will couple such manipulation with new sequencing technologies, proteomics or metabolomics to elucidate the molecular dialog between the different partners, as demonstrated in studies of host-symbiont and host-microbiome interactions (Chaston and Douglas, 2012; Rooks and Garrett, 2016). Development of these systems can provide a basis for developing new therapeutic and prevention strategies: vector hosts could be inoculated with defensive symbionts to reduce the transmission rate of a parasite; Parasite-associated microbes could be targeted to limit parasite development and allow its elimination by the host immune system; New parasite-specific probiotics could be selected to increase resistance or tolerance to parasitic infection. Developing these experimental systems for host-parasite-microbes’ interactions is now critical to determine which partner in the interaction should be targeted for effective therapy. Finally, investigating the role of microbes and microbiomes in host-parasite interactions may provide opportunities to confirm or infirm the hologenome theory of evolution by demonstrating multi-level selection.

**BOX 1 | Key Criteria for experimental model evaluation.**

(1) ** The host and parasite could be bred and maintained in the laboratory**. It is a necessary step to perform controlled experimental infections and understand the transmission mechanisms of symbionts of interest. It is also crucial for testing the environmental and host genetic factors that regulate the abundance, diversity and stability of the microbiome.
(2) ** A core microbiome would have been identified in the host** suggesting that it selects for specific microbes. This in itself suggests that the host should be considered as a holobiont.
(3) ** The parasite shares the same environment as microbes in the host body**, which would suggest potential direct parasite-host-associated microbe interaction that remain to be tested.
(4) ** The parasite would be associated with microbes** for which the role in virulence will be assessed. The presence of a core microbiome in parasites and the role of parasite-associated microbes in driving the evolution of host defenses remain to be demonstrated.
(5) ** Techniques have been developed to manipulate the composition of the microbiome of the host and/or parasite**. The role of microbes in the result of infection can only be tested through manipulation of the microbiome and injection of isolated microbes. It is necessary to characterize the role of microbes individually and as a community in susceptibility, resistance, and infectivity.
(6) ** The host and the parasite would have a wide geographical species range and ecological diversity**, which would allow comprehensive fields studies to understand how environmental factors influence host-parasite-microbe interaction. It would allow to examine the role of microbes in local adaptation of parasites through cross-infection experiments.
(7) ** The host and/or parasite would have short generation time**, which would facilitate the use experimental evolution to test the role of microbes in host or parasite adaptation. For instance, the hologenome theory would predict that associated microbes participate in the arms race through shorter generation time and higher mutation rate but this hypothesis remains to be tested.
(8) ** Closely related species of hosts could be parasitized with closely related species of parasites**, thus allowing comparative studies of the role of microbes in the interaction and testing their role during parasite evolution, host switch, and host or parasite speciation.
(9) ** The parasite would have various effects on the phenotype of its host**, so that the role of microbes in any aspect of the ’normal’ behavior or appearance of infected organism may be more readily detected and more reliably assessed for fitness consequences.
(10) ** The genome of the host and parasite would have been sequenced**, facilitating the use of high throughput comparative genomics approaches to characterize the genes involved in

## Author Contributions

All authors listed, have made substantial, direct and intellectual contribution to the work, and approved it for publication.

## Conflict of Interest Statement

The authors declare that the research was conducted in the absence of any commercial or financial relationships that could be construed as a potential conflict of interest.
