# Supplementary material for: Experimental Models to Study the Role of Microbes in Host-Parasite Interactions
Source: Front Microbiol. 2016 Aug 23;7:1300. doi: 10.3389/fmicb.2016.01300 (PMC4993751; doi:10.3389/fmicb.2016.01300)
Supplement: Supplementary file 1 [file Table_1.DOCX]

| **Host (s)** | **Parasite (s)** | **Host-associated microbe(s)** | **Parasite-associated microbes(s)** | **Genome available (Y/N) for H, P, M** | **Genome reference** | **Studies** | **Criteria Satisfied** |
| --- | --- | --- | --- | --- | --- | --- | --- |
| **Host defensive Symbiosis** | | | | | | | |
| Plethodon cinereus | *Batrachochytrium dendrobatidis* | Cutaneous bacteria | - | N(H), Y(P), N(M) | (Kuo et al., 2009) | (Brucker et al., 2007; Brucker et al., 2008) | 1, 3, 5, 6, 10 |
| Upupa epops, Phoeniculus purpureus | External predators and parasites | *Enterococcus faecalis* | - | N(H), N(P), N(M) | - | (Martín-Platero et al., 2006; Ruiz-Rodriguez et al., 2009; Ruiz-Rodríguez et al., 2013; Martín‐Vivaldi et al., 2014) | 3, 6, 8 |
| Acyrthosiphon pisum | *Aphidius ervi, Pandora neoaphidis* | *Hamiltonella, Serratia, Regiella* | - | Y(H), N(P), Y(M) | (Degnan et al., 2009) | (van der Wilk et al., 1999; Oliver et al., 2003; Oliver et al., 2005; Scarborough et al., 2005; Oliver et al., 2008; Weldon et al., 2013; Oliver et al., 2014; Polin et al., 2014; Martinez et al., 2016) | 1, 2, 3, 5, 6, 7, 8, 10 |
| Aedes aegypti | Dengue virus | *Wolbachia* | - | Y(H), Y(P), N(M) | (Osatomi and Sumiyoshi, 1990; Nene et al., 2007) | (Moreira et al., 2009; Bian et al., 2010; Van den Hurk et al., 2012) | 1, 2, 3, 5, 6, 7, 8, 10 |
| Aphis fabae | *Lysiphlebus fabarum* | *Hamiltonella defensa* | - | N(H), N(P), Y(M) | (Degnan et al., 2009) | (Vorburger et al., 2013; Cayetano et al., 2015) | 1, 2, 3, 5, 6, 7, 8, 10 |
| Drosophilia melanogaster | *Drosophila* C viruses, Cricket Paralysis Virus, Flock house virus, Nora virus, West Nile Virus | *Wolbachia* | - | Y(H), Y(P), Y(M) | (Johnson and Christian, 1998; Adams et al., 2000; Wu et al., 2004) | (Hedges et al., 2008; Teixeira et al., 2008; Osborne et al., 2009; Glaser and Meola, 2010) | 1, 2, 3, 5, 6, 7, 8, 10 |
| Hypera postica | *Microctonius aethiopoides* | *Wolbachia* | - | N(H), N(P), N(M) | N/A | (Hsaio, 1996) | 1, 3, 5, 6, 7, 8 |
| **Putative-host Defensive Symbiosis** | | | | | | | |
| Humans | *Mycobacterium tuberculosis* | *Helicobacter pylori** | - | Y(H), Y(P), Y(M) | (Tomb et al., 1997; Venter et al., 2001) | (Perry et al., 2010) | 2, 3, 10 |
| Lab mice | *Listeria monocytogenes, Yersinia pestis* | Latent herpesvirus | - | Y(H), Y(P), N(M) | (Glaser et al., 2001; Parkhill et al., 2001; Waterston et al., 2002) | (Barton et al., 2007) | 1, 2, 3, 5, 10 |
| **Putative Biological Weapon Strategy** | | | | | | | |
| Humans | *Onchocerca volvulus* | - | *Wuchereria bancrofti* | Y(H), N(P), N(M) | (Venter et al., 2001) | (Francis et al., 1985; Hall and Pearlman, 1999; Dreyer et al., 2000; Saint André et al., 2002) | 2, 3, 4, 10 |
| Humans, Hamsters, and other mammals | *Opisthorchis viverrini* | Intestinal and biliary microbiome | Bifidobacteriaceae, Enterobacteriaceae, and Listeriaceae | Y(H)*, Y(P), N(M) | (Venter et al., 2001; Young et al., 2014) | (Plieskatt et al., 2013; Chng et al., 2016; Osborne and WegenerParfrey, 2016) | 1,2*, 3, 5*, 8*, 10 |
| Humans | *Trichomonas vaginalis* | - | *Trichomonavirus* | Y(H), Y(P), N(M) | (Venter et al., 2001; Carlton et al., 2007) | (Fichorova et al., 2012) | 2, 3, 4, 10 |
| Humans | *Leishmania* | - | *Leishmania* RNA Virus-1 | Y(H), Y(P), Y(M) | (Stuart et al., 1992; Venter et al., 2001; Ivens et al., 2005) | (Ives et al., 2011) | 2, 3, 4, 10 |
| Humans and other mammals | *Giarda* | - | RNA virus | Y(H), Y(P), N(M) | (McArthur et al., 2000; Venter et al., 2001) | (Wang and Wang, 1991) | 1*, 2, 3, 4, 5*, 10 |
| Humans | *Plasmodium* | - | RNA virus | Y(H), Y(P), Y(M) | (Venter et al., 2001; Gardner et al., 2002; Carlton, 2003) | (Wang and Wang, 1991) | 2, 3, 4, 10 |
| Humans and other mammals | *Entamoeba* | - | RNA virus | Y(H), Y(P), N(M) | (Venter et al., 2001; Loftus et al., 2005) | (Wang and Wang, 1991) | 1*, 2, 3, 4, 5*, 10 |
| Humans and other mammals | *Naegleria* | - | RNA virus | Y(H), Y(P), N(M) | (Venter et al., 2001; Zysset-Burri et al., 2014) | (Wang and Wang, 1991) | 1*, 2, 3, 4, 5*, 10 |
| Humans and other mammals | *Eimeria* | - | RNA virus | Y(H), Y(P), Y(M) | (Venter et al., 2001; Shirley et al., 2004; Fraga et al., 2006) | (Wang and Wang, 1991) | 1*, 2, 3, 4, 5*, 10 |
| Humans and other mammals | *Cryptosporidium* | - | RNA virus | Y(H), Y(P), N(M) | (Venter et al., 2001; Abrahamsen et al., 2004; Xu et al., 2004) | (Wang and Wang, 1991) | 1*, 2, 3, 4, 5*, 10 |
| Humans and other mammals | *Babesia* | - | RNA virus | Y(H), Y(P), N(M) | (Venter et al., 2001; Brayton et al., 2007; Cornillot et al., 2012; Jackson et al., 2014) | (Wang and Wang, 1991) | 1*, 2, 3, 4, 5*, 10 |
| Ladybeetles | *Dinocampus coccinellae* | *Flavobacteria Rickettsia, Spiroplasma, Wolbachia* | *D. coccinellae* paralysis virus (DcPV) | N(H), N(P), Y(M) | (Dheilly et al., 2015) | (Hurst et al., 1994; Hurst et al., 1997; Weinert et al., 2007; Elnagdy et al., 2013; Dheilly et al., 2015) | 1, 2, 3, 4, 5, 6, 7, 8, 9 |
| Freshwater mollusks | *Cotylogaster occidentalis* | - | Picornavirus | N(H), N(P), N(M) | - | (Ip and Desser, 1984) | 1*, 2*, 3, 4, 6, 8 |
| **Putative Parasite Disruptive Strategy** | | | | | | | |
| Cattle | *Ostertagia ostertagi* | Gut microbiota | - | Y(H), N(P), N(M) | (Zimin et al., 2009) | (Li et al., 2011) | 1, 2, 3, 5, 10 |
| Esox Lucius | *Triaenophorus nodulosus* | Gut bacteria | - | Y(H), N(P), N(M) | (Rondeau et al., 2014) | (Izvekova and Lapteva, 2004) | 1, 2, 3, 6, 8, 10 |
| Gasterosteus aculeatus | *Schistocephalus solidus* | Stickleback gut microbiome | - | Y(H), Y(P), N(M) | (Kingsley, 2003; Aslett and De Silva, 2014) | *In progress | 1, 2, 3, 4*, 5, 6, 8, 9, 10 |
| Goats | *Haemonchus contortus* | Abomasal microbiome | - | Y(H), Y(P), N(M) | (Dong et al., 2013; Schwarz et al., 2013) | (Li et al., 2016) | 1, 2, 3, 5, 8, 10 |
| Humans | *Necator americanus* | Gut microbiota | - | Y(H), Y(P), N(M) | (Venter et al., 2001; Tang et al., 2014) | (Cantacessi et al., 2014) | 2, 3, 4, 10 |
| Humans | Soil transmitted helminthes | Gut microbiota | - | Y(H), Y(P), N(M) | (Venter et al., 2001; Park et al., 2011; Foth et al., 2014; Jex et al., 2014) | (Lee et al., 2014) | 2, 3, 4, 10 |
| Lab mice | *Trichuris muris* | Healthy mouse microbiome | - | Y(H), Y(P), Y(M) | (Waterston et al., 2002; Foth et al., 2014; Xiao et al., 2015) | (Hayes et al., 2010; Kane et al., 2011; Kuss et al., 2011) | 1, 2, 3, 5, 10 |
| Locusta migratoria manilensis | *Paranosema locustae* | Gut microbiota | - | Y(H), N(P), N(M) | (Flook et al., 1995) | (Shi et al., 2014) | 1, 2, 3, 5, 6, 7, 8, 9, 10 |
| Lota lota | *Eubortium rugosum* | Gut bacteria | - | N(H), N(P), N(M) | - | (Izvekova and Lapteva, 2004) | 1, 2, 3, 6, 8 |
| Pigs | *Trichuris suis* | Colon microbiome | - | Y(H), Y(P), N(M) | (Drou and Caccamo, 2011) | (Li et al., 2012; Wu et al., 2012) | 1, 2, 3, 5, 10 |
| Rat | *Hymenolepis diminuta* | Gut microbiota | - | Y(H), Y(P), N(M) | (von Nickisch-Rosenegk et al., 2001; Gibbs et al., 2004) | (McKenney et al., 2015) | 1, 2, 3, 5, 10 |
| Sheep | *Haemonchus contortus* | Abomasal microbiome | - | Y(H), Y(P), N(M) | (Archibald et al., 2010; Schwarz et al., 2013) | (Nicholls, 1987) | 1, 2, 3, 5, 10 |
| Oculina patagonica | *Vibrio shiloi* | Zooxanthellae | - | N(H), N(P), N(M) | - | (Kushmaro et al., 1997; Kushmaro et al., 1998; Toren et al., 1998; Ben‐Haim et al., 1999; Banin et al., 2000; Kushmaro et al., 2001) | 1, 2, 3, 6 |

**Supplementary Table 1**. Table one includes all of the model systems included in the paper and some others of interest for host-parasite-microbe studies. Also included is the strategy they suit, genome references, studies focused on in this paper, and the criteria that the model satisfies. Please note that criteria marked with an * may be conditional for that model. Additionally, please note, that for certain microbes, while there may be genome sequences available for certain strains, the genome for the strains specific to that model have not yet been sequenced. Some criteria may not be listed for some models, however, further study can lead to more criteria being satisfied for many models.

**Supplementary Works Cited**

Abrahamsen, M.S., Templeton, T.J., Enomoto, S., Abrahante, J.E., Zhu, G., Lancto, C.A., et al. (2004). Complete genome sequence of the apicomplexan, Cryptosporidium parvum. *Science.* 304(5669)**,** 441-445. doi: 10.1126/science.1094786.

Adams, M.D., Celniker, S.E., Holt, R.A., Evans, C.A., Gocayne, J.D., Amanatides, P.G., et al. (2000). The genome sequence of Drosophila melanogaster. *Science.* 287(5461)**,** 2185-2195.

Archibald, A.L., Cockett, N.E., Dalrymple, B.P., Faraut, T., Kijas, J.W., Maddox, J.F., et al. (2010). The sheep genome reference sequence: a work in progress. *Anim Genet.* 41(5)**,** 449-453. doi: 10.1111/j.1365-2052.2010.02100.x.

Aslett, A.M., and De Silva, N. (2014). *Pathogen Informatics.*

Banin, E., Israely, T., Kushmaro, A., Loya, Y., Orr, E., and Rosenberg, E. (2000). Penetration of the coral-bleaching bacterium *Vibrio shiloi* into *Oculina patagonica*. *Appl. Environ. Micro.* 66(7)**,** 3031-3036.

Barton, E.S., White, D.W., Cathelyn, J.S., Brett-McClellan, K.A., Engle, M., Diamond, M.S., et al. (2007). Herpesvirus latency confers symbiotic protection from bacterial infection. *Nature.* 447(7142)**,** 326-329.

Ben‐Haim, Y., Banim, E., Kushmaro, A., Loya, Y., and Rosenberg, E. (1999). Inhibition of photosynthesis and bleaching of zooxanthellae by the coral pathogen *Vibrio shiloi*. *Appl. Environ. Micro.* 1(3)**,** 223-229.

Bian, G., Xu, Y., Lu, P., Xie, Y., and Xi, Z. (2010). The endosymbiotic bacterium *Wolbachia* induces resistance to dengue virus in *Aedes aegypti*. *PLoS. Pathog.* 6(4)**,** e1000833. doi: 10.1371/journal.ppat.1000833.

Brayton, K.A., Lau, A.O., Herndon, D.R., Hannick, L., Kappmeyer, L.S., Berens, S.J., et al. (2007). Genome sequence of Babesia bovis and comparative analysis of apicomplexan hemoprotozoa. *PLoS Pathog.* 3(10)**,** 1401-1413. doi: 10.1371/journal.ppat.0030148.

Brucker, R.M., Baylor, C.M., Walters, R.L., Lauer, A., Harris, R.N., and Minbiole, K.P.C. (2007). The identification of 2,4-diacetylphloroglucinol as an antifungal metabolite produced by cutaneous bacteria of the salamander *Plethodon cinereus*. *J. Chem. Ecol.* 34(1)**,** 39-43. doi: 10.1007/s10886-007-9352-8.

Brucker, R.M., Harris, R.N., Schwantes, C.R., Gallaher, T.N., Flaherty, D.C., Lam, B.A., et al. (2008). Amphibian chemical defense: antifungal metabolites of the microsymbiont *Janthinobacterium lividum* on the salamander *Plethodon cinereus*. *J. Chem. Ecol.* 34(11)**,** 1422-1429. doi: 10.1007/s10886-008-9555-7.

Cantacessi, C., Giacomin, P., Croese, J., Zakrzewski, M., Sotillo, J., McCann, L., et al. (2014). Impact of experimental hookworm infection on the human gut microbiota. *J. Infect. Dis.* 210(9)**,** 1431-1434.

Carlton, J. (2003). The Plasmodium vivax genome sequencing project. *Trends in parasitology.* 19(5)**,** 227-231.

Carlton, J.M., Hirt, R.P., Silva, J.C., Delcher, A.L., Schatz, M., Zhao, Q., et al. (2007). Draft genome sequence of the sexually transmitted pathogen Trichomonas vaginalis. *Science.* 315(5809)**,** 207-212. doi: 10.1126/science.1132894.

Cayetano, L., Rothacher, L., Simon, J.-C., and Vorburger, C. (2015). Cheaper is not always worse: strongly protective isolates of a defensive symbiont are less costly to the aphid host. *Proc. R. Soc. Lond. [Biol].* 282(1799)**,** 20142333.

Chng, K.R., Chan, S.H., Ng, A.H.Q., Li, C., Jusakul, A., Bertrand, D., et al. (2016). Tissue microbiome profiling identifies an enrichment of specific enteric bacteria In Opisthorchis viverrini associated cholangiocarcinoma. *EBioMedicine.*

Cornillot, E., Hadj-Kaddour, K., Dassouli, A., Noel, B., Ranwez, V., Vacherie, B., et al. (2012). Sequencing of the smallest Apicomplexan genome from the human pathogen Babesia microti. *Nucleic Acids Res.* 40(18)**,** 9102-9114. doi: 10.1093/nar/gks700.

Degnan, P.H., Yu, Y., Sisneros, N., Wing, R.A., and Moran, N.A. (2009). Hamiltonella defensa, genome evolution of protective bacterial endosymbiont from pathogenic ancestors. *Proc Natl Acad Sci.* 106(22)**,** 9063-9068. doi: 10.1073/pnas.0900194106.

Dheilly, N.M., Maure, F., Ravallec, M., Galinier, R., Doyon, J., Duval, D., et al. (2015). Who is the puppet master? Replication of a parasitic wasp-associated virus correlates with host behaviour manipulation. *Proc. R. Soc. Lond.* 282(1803). doi: 10.1098/rspb.2014.2773.

Dong, Y., Xie, M., Jiang, Y., Xiao, N., Du, X., Zhang, W., et al. (2013). Sequencing and automated whole-genome optical mapping of the genome of a domestic goat (Capra hircus). *Nat Biotechnol.* 31(2)**,** 135-141. doi: 10.1038/nbt.2478.

Dreyer, G., Noroes, J., Figueredo-Silva, J., and Piessens, W. (2000). Pathogenesis of lymphatic disease in Bancroftian filariasis: a clinical perspective. *Parasitol. Today.* 16(12)**,** 544-548.

Drou, N., and Caccamo, M. (2011). Sus scrofa, whole genome shotgun sequencing project. *Bioinformatics.*

Elnagdy, S., Messing, S., and Majerus, M.E. (2013). Two Strains of Male-Killing Wolbachia in a Ladybird, Coccinella undecimpunctata, from a Hot Climate. *PloS one.* 8(1)**,** e54218.

Fichorova, R.N., Lee, Y., Yamamoto, H.S., Takagi, Y., Hayes, G.R., Goodman, R.P., et al. (2012). Endobiont viruses sensed by the human host–beyond conventional antiparasitic therapy. *PLoS ONE.* 7(11)**,** e48418.

Flook, P.K., Rowell, C.H., and Gellissen, G. (1995). The sequence, organization, and evolution of the Locusta migratoria mitochondrial genome. *J Mol Evol.* 41(6)**,** 928-941.

Foth, B.J., Tsai, I.J., Reid, A.J., Bancroft, A.J., Nichol, S., Tracey, A., et al. (2014). Whipworm genome and dual-species transcriptome analyses provide molecular insights into an intimate host-parasite interaction. *Nat. genet.* 46(7)**,** 693-700.

Fraga, J., Katsuyama, A., Fernandez, S., Madeira, A., Briones, M., and Gruber, A. (2006). The genome of the Eimeria brunetti RNA virus 1 is more closely related to fungal than to protozoan viruses. *GenBank, Accession No. NC_002701.*

Francis, H., Awadzi, K., and Ottesen, E. (1985). The Mazzotti reaction following treatment of onchocerciasis with diethylcarbamazine: clinical severity as a function of infection intensity. *Am. J. Trop. M. Hyg.* 34(3)**,** 529-536.

Gardner, M.J., Hall, N., Fung, E., White, O., Berriman, M., Hyman, R.W., et al. (2002). Genome sequence of the human malaria parasite Plasmodium falciparum. *Nature.* 419(6906)**,** 498-511. doi: 10.1038/nature01097.

Gibbs, R.A., Weinstock, G.M., Metzker, M.L., Muzny, D.M., Sodergren, E.J., Scherer, S., et al. (2004). Genome sequence of the Brown Norway rat yields insights into mammalian evolution. *Nature.* 428(6982)**,** 493-521. doi: 10.1038/nature02426.

Glaser, P., Frangeul, L., Buchrieser, C., Rusniok, C., Amend, A., Baquero, F., et al. (2001). Comparative genomics of Listeria species. *Science.* 294(5543)**,** 849-852. doi: 10.1126/science.1063447.

Glaser, R.L., and Meola, M.A. (2010). The native *Wolbachia* endosymbionts of *Drosophila melanogaster* and *Culex quinquefasciatus* increase host resistance to West Nile virus infection. *PLoS ONE.* 5(8)**,** e11977. doi: 10.1371/journal.pone.0011977.

Hall, L.R., and Pearlman, E. (1999). Pathogenesis of onchocercal keratitis (river blindness). *Clin. Microbiol. Rev.* 12(3)**,** 445-453.

Hayes, K., Bancroft, A., Goldrick, M., Portsmouth, C., Roberts, I., and Grencis, R. (2010). Exploitation of the intestinal microflora by the parasitic nematode *Trichuris muris*. *Science.* 328(5984)**,** 1391-1394.

Hedges, L.M., Brownlie, J.C., O'Neill, S.L., and Johnson, K.N. (2008). *Wolbachia* and virus protection in insects. *Science.* 322(5902)**,** 702-702.

Hsaio, T. (1996). Studies of interactions between alfalfa weevil strains, *Wolbachia* endosymbionts and parasitoids. *Syst. Assoc. Spec. Vol. Ser.* 53**,** 51-72.

Hurst, G.D., Hammarton, T.C., Bandi, C., Majerus, T.M., Bertrand, D., and Majerus, M.E. (1997). The diversity of inherited parasites of insects: the male-killing agent of the ladybird beetle Coleomegilla maculata is a member of the Flavobacteria. *Genetical research.* 70(01)**,** 1-6.

Hurst, G.D., Purvis, E.L., Sloggett, J.J., and Majerus, M.E. (1994). The effect of infection with male-killing Rickettsia on the demography of female Adalia bipunctata L.(two spot ladybird). *Heredity.* 73(3)**,** 309-316.

Ip, H.S., and Desser, S.S. (1984). A picornavirus-like pathogen of *Cotylogaster occidentalis* (Trematoda: Aspidogastrea), an intestinal parasite of freshwater mollusks. *J. Invertebr. Pathol.* 43(2)**,** 197-206.

Ivens, A.C., Peacock, C.S., Worthey, E.A., Murphy, L., Aggarwal, G., Berriman, M., et al. (2005). The genome of the kinetoplastid parasite, Leishmania major. *Science.* 309(5733)**,** 436-442.

Ives, A., Ronet, C., Prevel, F., Ruzzante, G., Fuertes-Marraco, S., Schutz, F., et al. (2011). *Leishmania* RNA virus controls the severity of *Mucocutaneous leishmaniasis*. *Science.* 331(6018)**,** 775-778.

Izvekova, G., and Lapteva, N. (2004). Microflora associated with the digestive-transport surfaces of fish and their parasitic cestodes. *Russ. J. Ecol.* 35(3)**,** 176-180.

Jackson, A.P., Otto, T.D., Darby, A., Ramaprasad, A., Xia, D., Echaide, I.E., et al. (2014). The evolutionary dynamics of variant antigen genes in Babesia reveal a history of genomic innovation underlying host-parasite interaction. *Nucleic Acids Res.* 42(11)**,** 7113-7131. doi: 10.1093/nar/gku322.

Jex, A.R., Nejsum, P., Schwarz, E.M., Hu, L., Young, N.D., Hall, R.S., et al. (2014). Genome and transcriptome of the porcine whipworm Trichuris suis. *Nat Genet.* 46(7)**,** 701-706. doi: 10.1038/ng.3012.

Johnson, K.N., and Christian, P.D. (1998). The novel genome organization of the insect picorna-like virus Drosophila C virus suggests this virus belongs to a previously undescribed virus family. *Journal of General Virology.* 79(1)**,** 191-203.

Kane, M., Case, L.K., Kopaskie, K., Kozlova, A., MacDearmid, C., Chervonsky, A.V., et al. (2011). Successful transmission of a retrovirus depends on the commensal microbiota. *Science.* 334(6053)**,** 245-249.

Kingsley, D. (2003). Sequencing the genome of threespine sticklebacks (Gasterosteus aculeatus).

Kuo, A., Samov, A., Schmutz, J., Lucas, S., Pitluck, S., Rosenblum, E., et al. (2009). Batrachochytrium dendrobatidis JAM81, whole genome shotgun sequencing project. *US DOE Genome Joint Genome Institute.*

Kushmaro, A., Banin, E., Loya, Y., Stackebrandt, E., and Rosenberg, E. (2001). *Vibrio shiloi* sp. nov., the causative agent of bleaching of the coral *Oculina patagonica*. *Int. J. Syst. Evol. Microbiol.* 51(4)**,** 1383-1388.

Kushmaro, A., Rosenberg, E., Fine, M., Ben Haim, Y., and Loya, Y. (1998). Effect of temperature on bleaching of the coral *Oculina patagonica* by Vibrio AK-1. *Mar. Ecol. Prog. Ser.* 171**,** 131-137.

Kushmaro, A., Rosenberg, E., Fine, M., and Loya, Y. (1997). Bleaching of the coral *Oculina patagonica* by Vibrio AK-1. *Mar. Ecol. Prog. Ser.* 147(1)**,** 159-165.

Kuss, S.K., Best, G.T., Etheredge, C.A., Pruijssers, A.J., Frierson, J.M., Hooper, L.V., et al. (2011). Intestinal microbiota promote enteric virus replication and systemic pathogenesis. *Science.* 334(6053)**,** 249-252.

Lee, S.C., San Tang, M., Lim, Y.A., Choy, S.H., Kurtz, Z.D., Cox, L.M., et al. (2014). Helminth colonization is associated with increased diversity of the gut microbiota. *PLoS Negl. Trop. Dis.* 8(5)**,** e2880.

Li, R.W., Li, W., Sun, J., Yu, P., Baldwin, R.L., and Urban, J.F. (2016). The effect of helminth infection on the microbial composition and structure of the caprine abomasal microbiome. *Sci. Rep.* 6.

Li, R.W., Wu, S., Li, W., Huang, Y., and Gasbarre, L.C. (2011). Metagenome plasticity of the bovine abomasal microbiota in immune animals in response to *Ostertagia ostertagi* infection. *PloS One.* 6(9)**,** e24417.

Li, R.W., Wu, S., Li, W., Navarro, K., Couch, R.D., Hill, D., et al. (2012). Alterations in the porcine colon microbiota induced by the gastrointestinal nematode *Trichuris suis*. *Infect. Immun.* 80(6)**,** 2150-2157.

Loftus, B., Anderson, I., Davies, R., Alsmark, U.C., Samuelson, J., Amedeo, P., et al. (2005). The genome of the protist parasite Entamoeba histolytica. *Nature.* 433(7028)**,** 865-868. doi: 10.1038/nature03291.

Martín-Platero, A.M., Valdivia, E., Ruíz-Rodríguez, M., Soler, J.J., Martín-Vivaldi, M., Maqueda, M., et al. (2006). Characterization of antimicrobial substances produced by *Enterococcus faecalis* MRR 10-3, isolated from the uropygial gland of the hoopoe (*Upupa epops*). *Appl. Environ. Microbiol.* 72(6)**,** 4245-4249.

Martín‐Vivaldi, M., Soler, J.J., Peralta‐Sánchez, J.M., Arco, L., Martín‐Platero, A.M., Martínez‐Bueno, M., et al. (2014). Special structures of hoopoe eggshells enhance the adhesion of symbiont‐carrying uropygial secretion that increase hatching success. *J. Anim. Ecol.* 83(6)**,** 1289-1301.

Martinez, A.J., Kim, K.L., Harmon, J.P., and Oliver, K.M. (2016). Specificity of multi-modal aphid defenses against two rival parasitoids. *PloS one.* 11(5)**,** e0154670.

McArthur, A.G., Morrison, H.G., Nixon, J.E., Passamaneck, N.Q., Kim, U., Hinkle, G., et al. (2000). The Giardia genome project database. *FEMS Microbiol Lett.* 189(2)**,** 271-273.

McKenney, E.A., Williamson, L., Yoder, A.D., Rawls, J.F., Bilbo, S.D., and Parker, W. (2015). Alteration of the rat cecal microbiome during colonization with the helminth *Hymenolepis diminuta*. *Gut Micro.* 6(3)**,** 182-193. doi: 10.1080/19490976.2015.1047128.

Moreira, L.A., Iturbe-Ormaetxe, I., Jeffery, J.A., Lu, G., Pyke, A.T., Hedges, L.M., et al. (2009). A *Wolbachia* symbiont in *Aedes aegypti* limits infection with dengue, *Chikungunya*, and *Plasmodium*. *Cell.* 139(7)**,** 1268-1278.

Nene, V., Wortman, J.R., Lawson, D., Haas, B., Kodira, C., Tu, Z.J., et al. (2007). Genome sequence of Aedes aegypti, a major arbovirus vector. *Science.* 316(5832)**,** 1718-1723. doi: 10.1126/science.1138878.

Nicholls, C. (1987). Endoscopy, physiology and bacterial flora of sheep infected with abomasal nematodes.

Oliver, K.M., Campos, J., Moran, N.A., and Hunter, M.S. (2008). Population dynamics of defensive symbionts in aphids. *Proc. R. Soc. Lond. [Biol].* 275(1632)**,** 293-299.

Oliver, K.M., Moran, N.A., and Hunter, M.S. (2005). Variation in resistance to parasitism in aphids is due to symbionts not host genotype. *Proc. Natl. Acad. Sci.* 102(36)**,** 12795-12800.

Oliver, K.M., Russell, J.A., Moran, N.A., and Hunter, M.S. (2003). Facultative bacterial symbionts in aphids confer resistance to parasitic wasps. *Proc. Natl. Acad. Sci.* 100(4)**,** 1803-1807.

Oliver, K.M., Smith, A.H., and Russell, J.A. (2014). Defensive symbiosis in the real world–advancing ecological studies of heritable, protective bacteria in aphids and beyond. *Funct. Ecol.* 28(2)**,** 341-355.

Osatomi, K., and Sumiyoshi, H. (1990). Complete nucleotide sequence of dengue type 3 virus genome RNA. *Virology.* 176(2)**,** 643-647.

Osborne, L.C., and WegenerParfrey, L. (2016). Liver Flukes and the Microbiota in Cancer. *EBioMedicine.*

Osborne, S.E., Leong, Y.S., O'Neill, S.L., and Johnson, K.N. (2009). Variation in antiviral protection mediated by different *Wolbachia* strains in *Drosophila simulans*. *PLoS Pathog.* 5(11)**,** e1000656. doi: 10.1371/journal.ppat.1000656.

Park, Y.C., Kim, W., and Park, J.K. (2011). The complete mitochondrial genome of human parasitic roundworm, Ascaris lumbricoides. *Mitochondr DNA.* 22(4)**,** 91-93. doi: 10.3109/19401736.2011.624608.

Parkhill, J., Wren, B.W., Thomson, N.R., Titball, R.W., Holden, M.T., Prentice, M.B., et al. (2001). Genome sequence of Yersinia pestis, the causative agent of plague. *Nature.* 413(6855)**,** 523-527. doi: 10.1038/35097083.

Perry, S., de Jong, B.C., Solnick, J.V., Sanchez, M.d.l.L., Yang, S., Lin, P.L., et al. (2010). Infection with *Helicobacter pylori* is associated with protection against tuberculosis. *PLoS ONE.* 5(1)**,** e8804. doi: 10.1371/journal.pone.0008804.

Plieskatt, J.L., Deenonpoe, R., Mulvenna, J.P., Krause, L., Sripa, B., Bethony, J.M., et al. (2013). Infection with the carcinogenic liver fluke *Opisthorchis viverrini* modifies intestinal and biliary microbiome. *FASEB. J.* 27(11)**,** 4572-4584.

Polin, S., Simon, J.C., and Outreman, Y. (2014). An ecological cost associated with protective symbionts of aphids. *Ecol. evol.* 4(6)**,** 836-840.

Rondeau, E.B., Minkley, D.R., Leong, J.S., Messmer, A.M., Jantzen, J.R., von Schalburg, K.R., et al. (2014). The genome and linkage map of the northern pike (Esox lucius): conserved synteny revealed between the salmonid sister group and the Neoteleostei. *PLoS One.* 9(7)**,** e102089. doi: 10.1371/journal.pone.0102089.

Ruiz-Rodríguez, M., Martínez-Bueno, M., Martín-Vivaldi, M., Valdivia, E., and Soler, J.J. (2013). Bacteriocins with a broader antimicrobial spectrum prevail in enterococcal symbionts isolated from the hoopoe's uropygial gland. *FEMS. Microbiol. Ecol.* 85(3)**,** 495-502.

Ruiz-Rodriguez, M., Valdivia, E., Soler, J.J., Martin-Vivaldi, M., Martin-Platero, A., and Martinez-Bueno, M. (2009). Symbiotic bacteria living in the hoopoe's uropygial gland prevent feather degradation. *J. Exp. Biol.* 212(22)**,** 3621-3626.

Saint André, A.v., Blackwell, N.M., Hall, L.R., Hoerauf, A., Brattig, N.W., Volkmann, L., et al. (2002). The role of endosymbiotic *Wolbachia* bacteria in the pathogenesis of river blindness. *Science.* 295(5561)**,** 1892-1895.

Scarborough, C.L., Ferrari, J., and Godfray, H. (2005). Aphid protected from pathogen by endosymbiont. *Science.* 310(5755)**,** 1781-1781.

Schwarz, E.M., Korhonen, P.K., Campbell, B.E., Young, N.D., Jex, A.R., Jabbar, A., et al. (2013). The genome and developmental transcriptome of the strongylid nematode Haemonchus contortus. *Genome Biol.* 14(8)**,** R89. doi: 10.1186/gb-2013-14-8-r89.

Shi, W., Guo, Y., Xu, C., Tan, S., Miao, J., Feng, Y., et al. (2014). Unveiling the mechanism by which microsporidian parasites prevent locust swarm behavior. *Proc. Natl. Acad. Sci.* 111(4)**,** 1343-1348. doi: 10.1073/pnas.1314009111.

Shirley, M.W., Ivens, A., Gruber, A., Madeira, A.M., Wan, K.-L., Dear, P.H., et al. (2004). The Eimeria genome projects: a sequence of events. *Trends in parasitology.* 20(5)**,** 199-201.

Stuart, K.D., Weeks, R., Guilbride, L., and Myler, P.J. (1992). Molecular organization of Leishmania RNA virus 1. *Proceedings of the National Academy of Sciences.* 89(18)**,** 8596-8600.

Tang, Y.T., Gao, X., Rosa, B.A., Abubucker, S., Hallsworth-Pepin, K., Martin, J., et al. (2014). Genome of the human hookworm Necator americanus. *Nat Genet.* 46(3)**,** 261-269. doi: 10.1038/ng.2875.

Teixeira, L., Ferreira, Á., and Ashburner, M. (2008). The bacterial symbiont *Wolbachia* induces resistance to RNA viral infections in *Drosophila* melanogaster. *PLoS Biol.* 6(12)**,** e1000002.

Tomb, J.F., White, O., Kerlavage, A.R., Clayton, R.A., Sutton, G.G., Fleischmann, R.D., et al. (1997). The complete genome sequence of the gastric pathogen Helicobacter pylori. *Nature.* 388(6642)**,** 539-547. doi: 10.1038/41483.

Toren, A., Landau, L., Kushmaro, A., Loya, Y., and Rosenberg, E. (1998). Effect of temperature on adhesion of *Vibrio* strain AK-1 to *Oculina patagonica* and on coral bleaching. *Appl. Environ. Microbiol.* 64(4)**,** 1379-1384.

Van den Hurk, A.F., Hall-Mendelin, S., Pyke, A.T., Frentiu, F.D., McElroy, K., Day, A., et al. (2012). Impact of *Wolbachia* on infection with chikungunya and yellow fever viruses in the mosquito vector *Aedes aegypti*. *PLoS Negl. Trop. Dis.* 6(11)**,** e1892.

van der Wilk, F., Dullemans, A.M., Verbeek, M., and van den Heuvel, J.F.J.M. (1999). Isolation and characterization of APSE-1, a bacteriophage infecting the secondary endosymbiont of *Acyrthosiphon pisum*. *Virol. J.* 262(1)**,** 104-113. doi: dx.doi.org/10.1006/viro.1999.9902.

Venter, J.C., Adams, M.D., Myers, E.W., Li, P.W., Mural, R.J., Sutton, G.G., et al. (2001). The sequence of the human genome. *Science.* 291(5507)**,** 1304-1351.

von Nickisch-Rosenegk, M., Brown, W.M., and Boore, J.L. (2001). Complete sequence of the mitochondrial genome of the tapeworm Hymenolepis diminuta: gene arrangements indicate that Platyhelminths are Eutrochozoans. *Mol Biol Evol.* 18(5)**,** 721-730.

Vorburger, C., Ganesanandamoorthy, P., and Kwiatkowski, M. (2013). Comparing constitutive and induced costs of symbiont‐conferred resistance to parasitoids in aphids. *Ecol. evol.* 3(3)**,** 706-713.

Wang, A., and Wang, C. (1991). Viruses of the protozoa. *Annu. Rev. Microbiol.* 45(1)**,** 251-263.

Waterston, R.H., Lindblad-Toh, K., Birney, E., Rogers, J., Abril, J.F., Agarwal, P., et al. (2002). Initial sequencing and comparative analysis of the mouse genome. *Nature.* 420(6915)**,** 520-562. doi: 10.1038/nature01262.

Weinert, L.A., Tinsley, M.C., Temperley, M., and Jiggins, F.M. (2007). Are we underestimating the diversity and incidence of insect bacterial symbionts? A case study in ladybird beetles. *Biology Letters.* 3(6)**,** 678-681.

Weldon, S.R., Strand, M.R., and Oliver, K.M. (2013). Phage loss and the breakdown of a defensive symbiosis in aphids. *Proc. R. Soc. Lond. [Biol].* 280(1751). doi: 10.1098/rspb.2012.2103.

Wu, M., Sun, L.V., Vamathevan, J., Riegler, M., Deboy, R., Brownlie, J.C., et al. (2004). Phylogenomics of the reproductive parasite Wolbachia pipientis wMel: a streamlined genome overrun by mobile genetic elements. *PLoS Biol.* 2(3)**,** E69. doi: 10.1371/journal.pbio.0020069.

Wu, S., Li, R.W., Li, W., Beshah, E., Dawson, H.D., and Urban Jr, J.F. (2012). Worm burden-dependent disruption of the porcine colon microbiota by *Trichuris suis* infection. *PloS One.* 7(4)**,** e35470.

Xiao, L., Feng, Q., Liang, S., Sonne, S.B., Xia, Z., Qiu, X., et al. (2015). A catalog of the mouse gut metagenome. *Nature Biotechnology.* 33(10)**,** 1103-1108.

Xu, P., Widmer, G., Wang, Y., Ozaki, L.S., Alves, J.M., Serrano, M.G., et al. (2004). The genome of Cryptosporidium hominis. *Nature.* 431(7012)**,** 1107-1112. doi: 10.1038/nature02977.

Young, N.D., Nagarajan, N., Lin, S.J., Korhonen, P.K., Jex, A.R., Hall, R.S., et al. (2014). The Opisthorchis viverrini genome provides insights into life in the bile duct. *Nat Commun.* 5**,** 4378. doi: 10.1038/ncomms5378.

Zimin, A.V., Delcher, A.L., Florea, L., Kelley, D.R., Schatz, M.C., Puiu, D., et al. (2009). A whole-genome assembly of the domestic cow, Bos taurus. *Genome Biol.* 10(4)**,** R42. doi: 10.1186/gb-2009-10-4-r42.

Zysset-Burri, D.C., Muller, N., Beuret, C., Heller, M., Schurch, N., Gottstein, B., et al. (2014). Genome-wide identification of pathogenicity factors of the free-living amoeba Naegleria fowleri. *BMC Genomics.* 15**,** 496. doi: 10.1186/1471-2164-15-496.
